# Effects of Perineural Dexamethasone and Dexmedetomidine on Neuromonitoring and Analgesia in Pediatric Scoliosis Surgery: A Randomized Controlled Trial

**DOI:** 10.1097/BRS.0000000000005399

**Published:** 2025-05-20

**Authors:** Malgorzata Reysner, Juliusz Huber, Tomasz Reysner, Piotr Janusz, Grzegorz Kowalski, Przemysław Daroszewski, Katarzyna Wieczorowska-Tobis, Tomasz Kotwicki

**Affiliations:** a Department of Palliative Medicine, Poznan University of Medical Sciences, Poznań, Poland; b Department of Pathophysiology of Locomotor Organs, Wiktor Dega Orthopaedic Institute, Poznan University of Medical Sciences, Poznań, Poland; c Department of Spine Disorders and Pediatric Orthopedics, Wiktor Dega Orthopaedic Institute, Poznan University of Medical Sciences, Poznań, Poland; d Department of Organization and Management in Health Care, Poznan University of Medical Sciences, Poznań, Poland

**Keywords:** erector spinae plane block, pediatric scoliosis surgery, motor-evoked potentials, neuromonitoring, dexamethasone, dexmedetomidine, postoperative analgesia, opioid-sparing anesthesia

## Abstract

**Study Design.:**

A randomized controlled trial.

**Objective.:**

To evaluate the effects of dexamethasone (DEX) and dexmedetomidine (DEM) as adjuvants to the erector spinae plane block (ESPB) on motor-evoked potential (MEP) recordings, postoperative analgesia, and hemodynamic stability in pediatric scoliosis surgery.

**Summary of Background Data.:**

Intraoperative neuromonitoring using MEPs is crucial for assessing spinal cord integrity during scoliosis surgery. The ESPB is widely used for postoperative pain management; however, its impact on neuromonitoring remains uncertain, especially when combined with perineural adjuvants.

**Methods.:**

Ninety pediatric patients undergoing scoliosis correction surgery were randomized into three groups: (1) Control (ESPB with 0.2% ropivacaine), (2) DEX (ropivacaine + 0.1 mg/kg DEX), and (3) DEM (ropivacaine + 0.1 µg/kg DEM). The primary outcome was time to first opioid analgesia. Secondary outcomes included total opioid consumption, postoperative pain scores, MEP amplitude and latency, transcranial electrical stimulation (TES) intensity required to evoke MEP, and hemodynamic stability.

**Results.:**

Both adjuvants significantly prolonged analgesia and reduced opioid consumption (*P*<0.0001). Pain scores (numerical rating scale) at 8, 12, 16, and 24 hours were lower in both adjuvant groups compared to the control. DEX was associated with the highest MEP amplitudes postsurgical correction and required lower TES intensity (*P*=0.04), indicating superior neuromonitoring conditions. DEM was linked to lower MEP amplitudes and increased incidence of bradycardia (11 patients), whereas intraoperative hypotension occurred in five DEX patients.

**Conclusions.:**

DEX improves neuromonitoring conditions by enhancing MEP amplitudes and reducing TES requirements, whereas DEM is associated with MEP suppression and hemodynamic instability. These findings highlight the importance of balancing analgesia with neuromonitoring integrity in pediatric scoliosis surgery.

Surgical correction of idiopathic scoliosis, particularly spinal fusion, is associated with significant postoperative pain due to extensive tissue dissection and spinal manipulation.^1^ Adequate postoperative analgesia is essential not only for patient comfort but also for early mobilization, minimization of opioid consumption, and prevention of chronic postoperative pain syndromes.^2^ A multimodal analgesic approach, integrating regional anesthesia techniques, has been increasingly employed to optimize pain management in scoliosis surgery.^3^

The erector spinae plane block (ESPB) has emerged as a promising regional analgesic technique because it provides both somatic and visceral analgesia.^4,5^ Its efficacy, relatively simple ultrasound-guided administration, and favorable safety profile have contributed to its growing use in spinal surgeries.^6^ Despite its widespread adoption, strategies to prolong the duration of ESPB-induced analgesia remain a subject of clinical investigation.^7^

The use of perineural adjuvants to extend the duration and quality of regional anesthesia has been explored.^8^ Among the most extensively studied agents are dexamethasone (DEX), a potent corticosteroid with anti-inflammatory properties, and dexmedetomidine (DEM), an α2-adrenergic agonist that modulates analgesia by inhibiting norepinephrine release and reducing sympathetic outflow.^9^ Both agents have demonstrated efficacy in peripheral nerve blocks,^9^ yet their comparative effectiveness in ESPB for major spinal surgeries, such as scoliosis correction, remains underexplored.

Given the increasing adoption of ESPB in scoliosis surgery and the necessity of optimizing pain management strategies, this study aims to directly compare DEX and DEM as perineural adjuvants in this clinical context. In addition, intraoperative neuromonitoring using motor-evoked potentials (MEPs) represents a warning component of scoliosis surgery, ensuring the integrity of spinal cord function throughout the procedure.^10^ While neuromonitoring techniques are well established, the impact of ESPB with different perineural adjuvants on intraoperative MEP parameters has not been investigated extensively enough. A systematic review by Reysner *et al.* ^11^ remains one of the few reports addressing the influence of anesthetic techniques on neuromonitoring during scoliosis surgery. However, the effect of adjuvant-enhanced ESPB on MEP recordings remains unexplored, necessitating further investigation into its implications for neurophysiological monitoring and surgical outcomes.

This randomized, double-blinded clinical trial aims to bridge this knowledge gap by evaluating the efficacy, safety, and neuromonitoring implications of DEX *versus* DEM as perineural adjuvants to ESPB in pediatric idiopathic scoliosis surgery.

## MATERIALS AND METHODS

### Study Design

This randomized controlled trial was conducted in Poland and was registered on ClinicalTrials.gov (NCT06086431) before the commencement of patient recruitment on October 11, 2023. Ethical approval was granted on September 13, 2023, by the Bioethics Committee at Poznan University of Medical Sciences, Poland (protocol number 538/23). Before enrollment, written informed consent was obtained from all patients’ caregivers. The recruitment period extended from October 17, 2023, to September 24, 2024. The study was conducted following the Declaration of Helsinki.

### Participants

Patients eligible for inclusion were children and adolescents aged between 10 and 18 who were scheduled to undergo surgery for idiopathic scoliosis. Only patients with Lenke type 2^12^ curves were included, as this subtype requires a relatively standardized surgical approach involving double thoracic curves. This allowed us to reduce variability in fusion levels and instrumentation extent, which could affect intraoperative neuromonitoring and postoperative pain outcomes. Exclusion criteria included infection at the regional block site, coagulation disorders, immunodeficiency, an ASA (American Society of Anesthesiologists) physical status classification of IV or higher, a history of epilepsy, the presence of implanted pacemakers or other electronic devices, and regular use of corticosteroids.

### Randomization and Blinding

**Figure 1:**
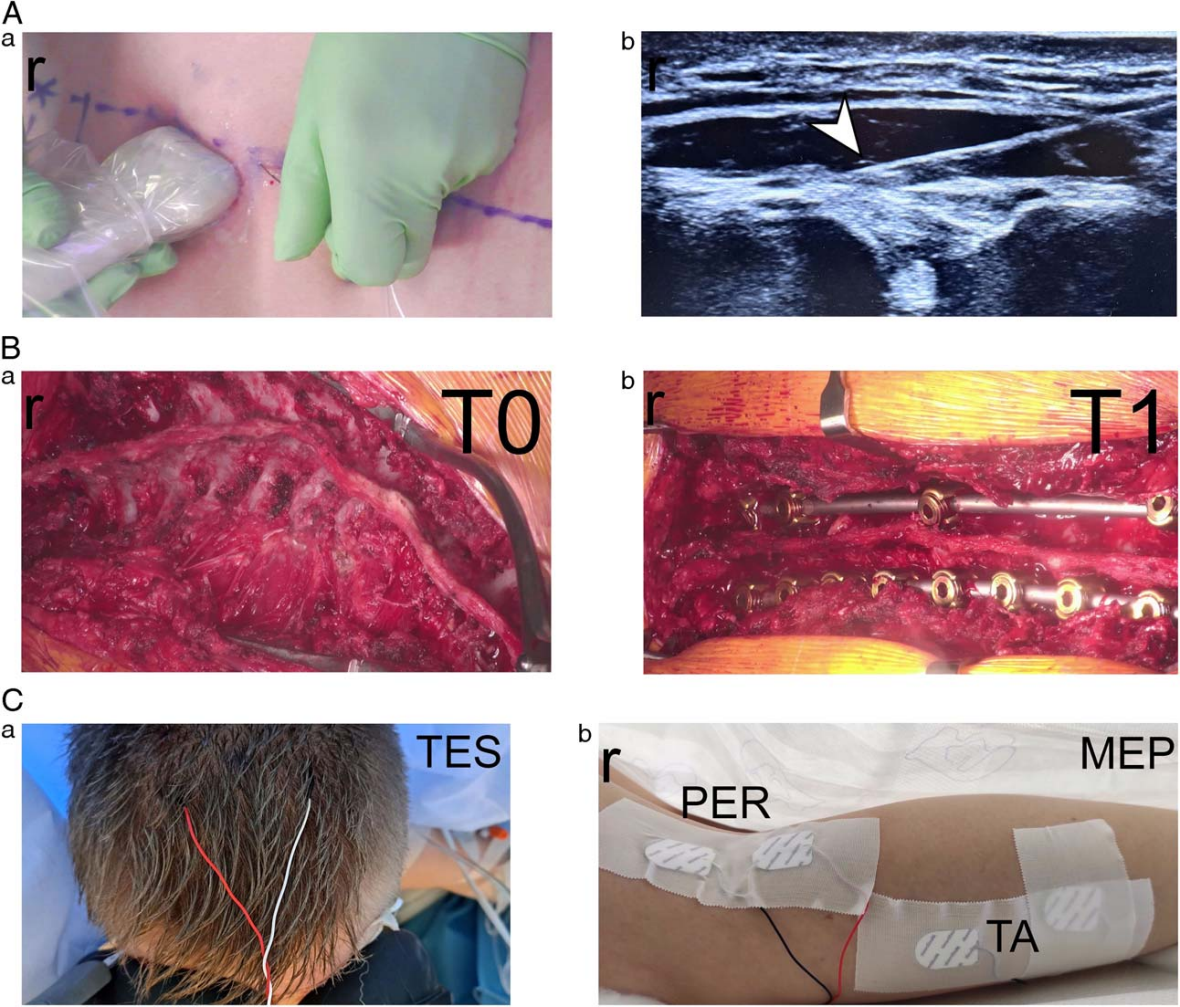
A, EPSB technique under ultrasonography control (a), the arrowhead indicates the location of the needle tip (b). B, Camera pictures of the surgical field before (a, T0) and after (b, T1) the implantation of the scoliosis corrective instrumentation with transpedicular screws and rods. C, Principles of the transcranial electrical stimulation (a, TES) with needle electrodes implanted over the scalp skin and surface electrodes placed bilaterally on tibialis anterior muscles (b, TA) and over the peroneal nerves (b, PER) for motor-evoked potential (MEP) recordings are presented in Figure 3. r indicates rostral direction.

A total of 90 patients diagnosed with Lenke type 2 idiopathic scoliosis^12^ were randomly assigned in a 1:1:1 ratio to one of three treatment groups: the control group, which received an ultrasound-guided ESPB (Figure 1A) with 0.2% ropivacaine; the DEX group, which received 0.2% ropivacaine with 0.1 mg/kg DEX; and the DEM group, which received 0.2% ropivacaine with 0.1 μg/kg DEM.

Although Lenke type 2 scoliosis is less common than Lenke type 1, our institution is a high-volume pediatric spine surgery center with a substantial referral base, making it feasible to enroll this number of Lenke 2 patients within a year.

The randomization sequence was generated using nQuery Advisor software (Statistical Solutions) through a computerized block randomization process with a six-block size to ensure balanced group allocation. Allocation was concealed in sequentially numbered, sealed opaque envelopes that were opened immediately before block administration. An anesthetic nurse not involved in the study prepared the trial medications and placed them in identical opaque syringes and sequentially numbered containers to maintain allocation concealment. The anesthesia team, surgical team, operating room staff, neurophysiologists, patients, and caregivers remained blinded to group allocation throughout the study. Unblinding occurred only after the completion of statistical analysis.

### Surgical Technique and Standardization

All surgical procedures were performed by a single specialized team at Wiktor Dega Orthopedic and Rehabilitation Hospital in Poznan, Poland. Spinal deformity correction was performed using convex rod rotation, apical translation, segmental derotation, concave-side distraction, and convex-side compression (Figure 1Bb). All patients underwent thoracic spinal fusion procedures involving vertebral levels T2 to T11. The average number of fused segments was 9.2 ± 1.1. No lumbar levels were included, and no posterior column osteotomies or vertebral column resections were performed. Surgical technique and extent were standardized across all patients. Spinal fusion was achieved through decortication, and pedicle screw placement was guided using intraoperative x-ray (C-arm) imaging and neuromonitoring. Patients were monitored for at least 48 hours postoperatively, with outcome data collected by independent researchers.

### Anesthetic Protocol

Preoperatively, all patients received oral midazolam (7.5 mg) as part of a multimodal preemptive analgesia regimen. General anesthesia was induced using intravenous (IV) propofol (2.5–3.5 mg/kg) and fentanyl (2 μg/kg), and endotracheal intubation was facilitated with rocuronium (0.6 mg/kg). Anesthesia was maintained with IV propofol (4–12 mg/kg/h) and remifentanil (0.5–1.5 μg/kg/min), titrated to maintain hemodynamic stability and a Bispectral Index (BIS) between 45 and 65 (GE Healthcare). Intraoperative analgesia was supplemented with IV acetaminophen (15 mg/kg), IV metamizole (15 mg/kg), and IV ibuprofen (10 mg/kg) to minimize opioid consumption.

### Regional Anesthesia: ESPB Administration

Following induction of anesthesia, bilateral, bilevel ESPBs (Figure 1A) were performed at Th4 and Th10 vertebral levels under ultrasound guidance (Mindray TE9). A 22-gauge Stimuplex Ultra 360, 80 mm needle was inserted using an in-plane technique with a linear ultrasound transducer, oriented caudally at Th4 and cranially at Th10. Proper needle placement within the erector spinae fascial plane was confirmed using hydro-location with 1 to 2 mL of 0.9% saline, followed by injection of 0.5 mL/kg of the assigned ESPB solution.

All ESP blocks were performed by one of two attending pediatric anesthesiologists with extensive experience in regional anesthesia, following a standardized protocol to ensure consistency across all patients. No significant deviations in technique were observed throughout the study. Notably, events such as bradycardia or respiratory depression were analyzed based on group allocation and showed no association with the individual block performer.

### Intraoperative Neuromonitoring

Intraoperative MEPs monitoring was conducted using the ISIS system (Inomed Medizintechnik) to ensure the integrity of motor function neural transmission during spinal deformity correction. MEPs were recorded bilaterally from the tibialis anterior (TA) (Figure 1Cb) muscles following transcranial electrical stimulation (TES) (Figure 1Ca). The amplitude and latency of MEPs were analyzed at two time points: T0 (baseline, before surgical incision) and T1 (following the implantation of pedicle screws and rods for spinal curvature correction), as seen in Figure 1Bb.

Transcranial stimulation was applied using a sequence of four electrical pulses, each lasting 500 µs and delivered at an average intensity of 98.2 mA. The stimulation was administered via bipolar subcutaneous needle electrodes, which were positioned according to the 10-20 international system at Cz–C3 (3–6 cm left) and Cz–C4 (3–6 cm right). The impedance of scalp electrodes was maintained between 0.6 and 0.8 kΩ to ensure optimal signal conduction.

MEP responses were recorded using surface disposable Ag/AgCl electrodes (5 mm^2^ active surface area) placed bilaterally over the TA muscles and peroneal (PER) nerves, as seen in Figure 1Cb. A sterile needle ground electrode was inserted at the iliac crest. Stimulation and recording electrodes were initially positioned and tested while the patient was supine, and the first MEP recordings were obtained as baseline reference values. After the patient was repositioned into the prone position, MEPs were continuously recorded at key intraoperative stages, including before and after spinal instrumentation (T0 and T1 observation periods).

The recorded MEPs demonstrated amplitudes ranging from 100 to 2000 µV and latencies between 27 and 40 ms. Averaging was not required due to the reliability of the recorded signals. The MEP recording system was configured with the following parameters: hardware high-pass filter at 30 Hz, software high-pass filter at 0.5 Hz, software low-pass filter at 2000 Hz, and stimulation frequency ranging from 0.5 to 2.4 ms intervals. These settings ensured high-fidelity signal acquisition and facilitated the assessment of motor pathway integrity during scoliosis surgery.

### Postoperative Pain Management

Postoperative analgesia was administered using a multimodal regimen consisting of IV acetaminophen (15 mg/kg every 6 h), IV metamizole (15 mg/kg every 6 h), and IV ibuprofen (10 mg/kg every 6 h). If the numerical rating scale (NRS) score exceeded 4, patients received IV morphine sulfate (0.1 mg/kg) as a rescue analgesic, followed by patient-controlled analgesia with morphine (0.2 mg/kg/h).

Pain intensity was evaluated at 4, 8, 12, 16, 20, and 24 hours postoperatively using an NRS scale (0=no pain, 10=worst pain imaginable). Total opioid consumption and time to first opioid administration were recorded.

### Outcome Assessment

The outcome assessment was conducted by two clinicians unaware of the group allocation.

#### Primary Outcomes

The time to first rescue opioid analgesia was evaluated by residents not involved in the study from the postoperative and orthopedic wards.

#### Secondary Outcomes

Total opioid consumption was assessed from the postoperative and orthopedic ward records by residents not involved in the study. The pain score was assessed using the NRS score (0 meaning no pain and 10 meaning the worst pain imaginable) at all postoperative time points (4, 8, 12, 16, 20, and 24 h after surgery). Two independent physicians examined each subject, and the final score was agreed upon at the end of the assessment.

Blood samples for platelet-to-lymphocyte ratio (PLR), neutrophil-to-lymphocyte ratio (NLR), and blood glucose were obtained 24 and 48 hours after surgery. Two researchers who were blinded to the group allocation assessed these outcomes.

All three groups recorded changes in TES and MEP parameters from the TA muscle before and after the surgical correction procedures (Figure 1C).

Nerve injuries/deficits were defined as follows: 0—no nerve damage, 1—minor sensory paresthesias, 2—complete sensory anesthesia, 3—complete motor defect with or without sensory paresthesia, and 4—Complex Regional Pain Syndrome.

Adverse effects were defined as bradycardia (<50 heartbeats/min during surgery), hypotension (30% or more decrease in mean arterial pressure from baseline values), nausea, and vomiting 48 hours after surgery. Two researchers who were blinded to the group allocation assessed these outcomes.

All neuromonitoring, adverse effects, and nerve injury data were collected at hospital discharge.

### Statistical Analysis

The sample size was based on our primary hypothesis that the time to first rescue opioid analgesia would be significantly longer in the DEX than in the DEM group. Our null hypothesis was that there would be no significant difference in the time to first rescue analgesia between these two groups. The time to first rescue opioid analgesia was the primary variable. Based on a pilot study on 10 patients not included in the final analysis, the time to first rescue opioid analgesia was 12.95 ± 1.68 hours (mean ± SD) in the DEX group and 11.22 ± 1.87 hours (mean ± SD) in the DEM group. Using pairwise comparison, we calculated the sample size required to detect a difference in the time to first rescue opioid analgesia among the three groups. Bonferroni correction was performed to adjust for the increased type I error rate in multiple comparisons. Accordingly, 28 subjects were required in each group to achieve a statistical power of 95% at a *P* value of <0.05. To facilitate block randomization and account for loss to follow-up, 30 pediatric patients per group (90 in total) were recruited.

The statistical analysis was performed using GraphPad Prism 10.1.1 (270) software from GraphPad Software Inc. in San Diego, CA. We used the Shapiro-Wilk normality test to evaluate the parametric distribution of numerical variables. Differences between groups were assessed using ANOVA with the post hoc Tukey test for numerical variables and the Kruskal-Wallis test for categorical variables. Contingency analysis between groups was conducted using the Fisher exact test. A *P* value of <0.05 was considered statistically significant and calculated with 95% CIs.

## RESULTS

### Summary of Participation

Out of 111 children assessed for eligibility, 13 were excluded for not meeting the inclusion criteria, and the caregivers of five children declined participation. This left 93 children who were randomly allocated into three groups. One patient in the control group did not receive the intervention due to the surgeon’s refusal postrandomization, and another from the control group, along with one patient from the DEX group, were lost to follow-up after requiring ICU admission for invasive mechanical ventilation. These ICU admissions were unrelated to ESP block administration. No block-related complications occurred in any patient, such as pneumothorax, local anesthetic systemic toxicity, or neurological injury.

**Figure 2:**
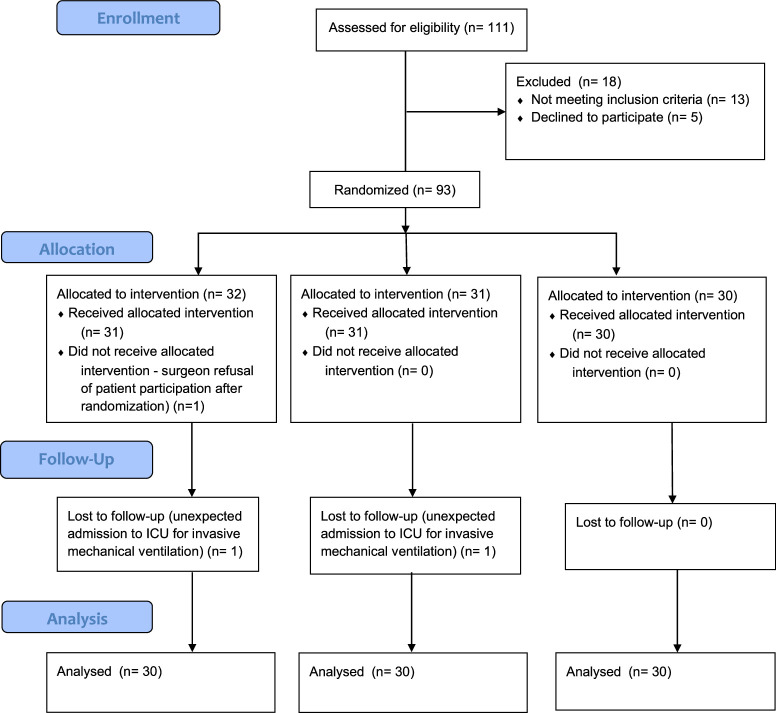
CONSORT flow diagram.

Ninety participants completed the study and were included in the final analysis (Figure 2). The baseline characteristics among the groups are presented in Table 1.

**TABLE 1:** Baseline Characteristics of Patients

Parameter	Control (Mean±SD or Count)	DEX (Mean±SD or Count)	DEM (Mean±SD or Count)	*P*
Age (yr)	14.3 (11–17)	14.6 (13–17)	14.7 (11–17)	0.4054
Weight (kg)	52.9 (34–63)	51.0 (42–64)	52.6 (42–62)	0.3328
Height (cm)	155.0 (148–182)	155.5 (148–169)	155.4 (147–163)	0.9246
Male/female	12/18	8/22	7/23	0.4377
Cobb’s angle (primary, degrees)	56.3±4.8 (40–89)	56.9±4.0 (42–86)	56.2±4.3 (42–85)	0.4430
Cobb’s angle (secondary, degrees)	36.5±3.4 (29–50)	37.1±3.8 (30–49)	36.9±3.6 (30–48)	0.1163
Time of surgery (min)	241.7±30.6 (175–300)	246.5±35.1 (175–295)	262.5±44.7 (175–355)	0.2110
Fusion levels	Th2–Th11	Th2–Th11	Th2–Th11	—
Number of fused segments	9.3±1.1	9.1±1.2	9.2±1.1	0.1582
Posterior column osteotomies	0	0	0	—
Vertebral column resections	0	0	0	—
NLR before surgery	1.44±0.55 (0.26–2.43)	1.65±0.60 (0.65–2.98)	1.38±0.63 (0.24–2.87)	0.2124
PLR before surgery	154.1±51.92 (43.54–232.1)	141.1±57.46 (72.29–222.7)	146.4±51.25 (45.68–222.3)	0.3152
ASA I	12	10	13	0.8245
ASA II	17	17	15	
ASA III	1	3	2	

Data are expressed as a number or mean (SD).

ASA indicates American Society of Anesthesiologists; DEM, dexmedetomidine; DEX, dexamethasone; NLR, neutrophile-to-lymphocyte ratio; PLR, platelet-to-lymphocyte ratio.

### Primary Outcomes

The time to first rescue opioid analgesia was significantly prolonged in both the DEX and DEM groups compared to the control group, as shown in Table 2A.

**TABLE 2:** Analgesic, Neuromonitoring, Laboratory, and Adverse Outcomes

Outcome	Control	DEX	DEM	*P* (^*^,^†^)	*P* (^‡^,^§^)	*P* (^§^,^‖^)	*P* (^§^,^¶^)
(A) Analgesic outcomes							
Time to first rescue opioid analgesia (h)	5.15±1.69	12.96±2.03	11.65±2.95	**<0.0001**	**<0.0001**	**<0.0001**	0.3975
Total opioid consumption (IV morphine equivalents, mg/kg)	3.06±0.84	2.12±0.42	2.13±0.31	**<0.0001**	**<0.0001**	**<0.0001**	>0.9999
Total opioid consumption (entire hospitalization, mg/kg)	4.90±1.15	3.58±0.81	3.63±0.74	<0.0001	<0.0001	<0.0001	>0.9999
NRS Pain Scores (4 h postoperatively)	1.97±0.56	1.70±0.47	1.73±0.45	0.1140	**0.0072**	**0.0157**	>0.9999
NRS Pain Scores (8 h postoperatively)	2.13±0.63	1.67±0.48	1.70±0.47	**0.0034**	**0.0006**	**0.0001**	>0.9999
NRS Pain Scores (12 h postoperatively)	2.23±0.68	1.57±0.50	1.50±0.51	**<0.0001**	**<0.0001**	**<0.0001**	>0.9999
NRS Pain Scores (16 h postoperatively)	2.33±0.55	1.57±0.50	1.60±0.50	**<0.0001**	**0.0260**	0.8241	0.3760
NRS Pain Scores (20 h postoperatively)	2.03±0.56	1.77±0.50	1.97±0.56	**0.1483**	—	—	—
NRS Pain Scores (24 h postoperatively)	2.20±0.55	1.80±0.55	2.03±0.61	0.0309	—	—	—
(B) Neuromonitoring, laboratory, and adverse outcomes							
Bradycardia during surgery (<50/min)	0	0	11	**<0.0001**	—	—	—
Hypotension during surgery (>30% decrease in MAP)	0	0	5	**<0.0001**	—	—	—
Neutrophil-to-lymphocyte ratio (24 h)	2.06±1.59	1.76±0.75	1.65±0.80	0.3367	—	—	—
Neutrophil-to-lymphocyte ratio (48 h)	2.21±0.56	1.98±0.50	2.02±0.59	0.2130	—	—	—
Platelet-to-lymphocyte ratio (24 h)	139.5±58.14	129.9±59.75	163.5±45.55	0.0562	—	—	—
Platelet-to-lymphocyte ratio (48 h)	202.6±60.27	203.8±61.11	181.1±51.54	0.2430	—	—	—
Blood glucose (12 h postoperatively)	87.10±7.63	85.50±7.23	86.53±4.97	0.6744	—	—	—
Blood glucose (24 h postoperatively)	85.97±6.36	85.87±6.92	85.13±6.52	0.8273	—	—	—
Blood glucose (48 h postoperatively)	86.87±6.22	84.87±7.12	86.07±5.96	0.5065	—	—	—
Nerve damage (12 h postoperatively)	0	0	0	—	—	—	—
Nerve damage (24 h postoperatively)	0	0	0	—	—	—	—
Nerve damage (48 h postoperatively)	0	0	0	—	—	—	—

Bold values indicate statistical significance.

Values are mean (SD) or number.

^*^ compared all three groups.

^†^ ANOVA test.

^‡^ *P* value compares the control group to the DEX.

^§^ ANOVA test with the post hoc Tukey’s test used to compare means between the groups or the Fisher exact test.

^‖^ compares the control group to the DEM.

^¶^ compares the DEX to DEM.

Comparison of analgesic effectiveness, opioid consumption, pain scores, hemodynamic events, and laboratory markers across study groups (Control, DEX, and DEM). Values are presented as mean (SD) or number.

DEM indicates dexmedetomidine; DEX, dexamethasone; IV, intravenous; MAP, mean arterial pressure; NLR, neutrophile-to-lymphocyte ratio; NRS, numeric rating scale; PLR, platelet-to-lymphocyte ratio.

### Secondary Outcomes

#### Total Opioid Consumption

The total opioid consumption during the first 24 hours (mg/kg in morphine equivalents) was significantly lower in the DEX (2.12 ± 0.42) and DEM (2.13 ± 0.31) groups compared with the control group (3.06 ± 0.84, *P* < 0.0001). No significant difference was observed between the DEX and DEM groups (*P* > 0.9999). When evaluating opioid use over the entire hospitalization period, the DEX (3.58 ± 0.81) and DEM (3.63 ± 0.74) groups again showed significantly reduced morphine-equivalent requirements compared with the control group (4.90 ± 1.15, *P* < 0.0001), with no difference between the DEX and DEM groups (*P* > 0.9999).

#### NRS Pain Scores

Postoperative pain scores (NRS) were significantly lower in the DEX and DEM groups at 8 hours (*P*=0.0034), 12 hours (*P*<0.0001), 16 hours (*P*<0.0001), and 24 hours (*P*=0.0309) compared with the control group. No significant differences were found between the DEX and DEM groups at any time point.

#### Inflammatory Markers and Metabolic Parameters

At any time point, no significant differences were observed among the groups for NLR, PLR, or blood glucose levels, as seen in Table 2B.

#### Neurological Safety

No cases of nerve injury were reported in any of the groups.

#### Adverse Effects

Intraoperative bradycardia occurred in 11 patients, and intraoperative hypotension was observed in five patients in the DEM group. No cases of intraoperative bradycardia or hypotension were observed in the DEX group. No cases of nausea or vomiting were reported in any of the groups.

### Neuromonitoring

**Figure 3:**
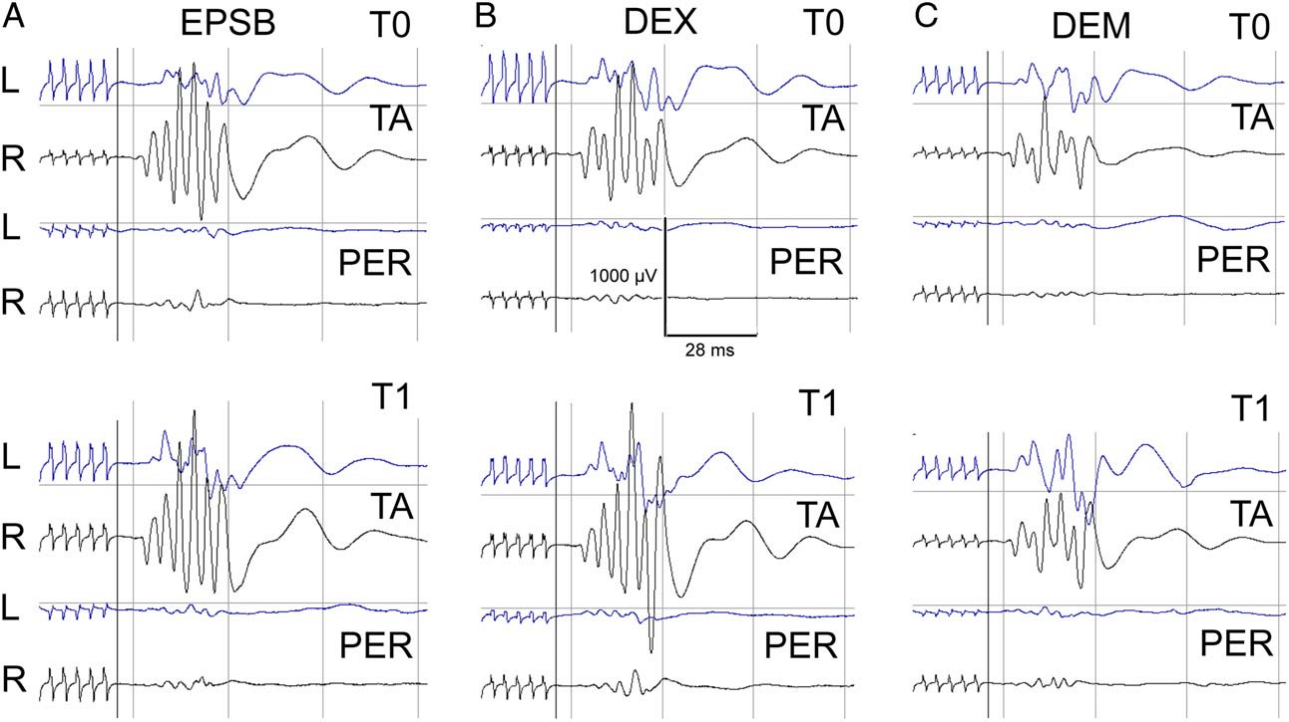
Examples of MEP recorded bilaterally from tibialis anterior (TA) peroneal nerves (PER) at T0 and T1 periods of observation in three scoliotic patients belonging to three examined groups (A, Control with EPSB; B, DEX; C, DEM). Calibration bars for amplification and time base are the same for all recordings. L indicates left; R, right.

Table 3 presents the comparative analysis of BIS values, TES parameters, and MEP recordings between the control, DEX, and DEM groups. The mean BIS values at baseline (T0) and postsurgical correction (T1) did not differ significantly between the DEX and DEM groups compared with the control group. Furthermore, these values remained consistent with normative reference data reported in previous literature.^13^ The mean TES stimulus intensity required for optimal MEP induction was significantly lower in the DEX group compared with the control group (*P*=0.04). A slight reduction in MEP latency was observed in the DEX and DEM groups compared with controls at T0 and T1, but these differences did not reach statistical significance. The mean MEP amplitude change between T0 and T1 was most pronounced in the DEX group (Figure 3B), with a significantly more significant increase compared with the control group (*P*=0.04).

**TABLE 3:** Neuromonitoring Parameters: BIS, TES, and MEP Measurements Before and After Surgical Correction

Parameter	Control	DEX	DEM	*P* (^*^,^†^)	*P* (^†^,^‡^)	*P* (^†^,^§^)
Mean BIS indicator	51.1±4.2	53.7±4.5	50.3±4.1	0.06	0.07	0.05
Mean TES stimulus strength (mA)	95.1±9.4	91.6±8.2	94.2±7.9	**0.04**	0.06	0.05
Mean MEP amplitude (µV) T0	410.4±55.4	409.3±51.2	412.3±52.1	0.08	0.07	0.06
Mean MEP amplitude (µV) T1	680.3±43.8	820.4±43.8	668.2±41.4	**0.04**	0.06	**0.04**
p (T0 *vs.* T1)	**0.04**	**0.02**	**0.04**	—	—	—
Mean MEP latency (ms) T0	31.9±2.2	32.0±1.9	31.5±2.0	0.07	0.09	0.06
Mean MEP latency (ms) T1	30.5±2.6	31.4±2.2	30.5±2.3	0.06	0.08	0.06
p (T0 *vs.* T1)	0.05	0.05	0.05	—	—	—

Bold values indicate statistical significance.

Values are mean (SD) or number.

^*^ *P* value compares the control group to the DEX.

^†^ ANOVA test with the post hoc Tukey test used to compare means between the groups or the Fisher exact test.

^‡^ Compares the control group to the DEM.

^§^ Compares the DEX to DEM.

BIS indicates Bispectral Index Monitor; DEM, dexmedetomidine; DEX, dexamethasone; MEP, TES-induced motor-evoked potential; T0, before surgical correction; T1, after surgical correction; TES, transcranial electrical stimulation.

## DISCUSSION

The findings of this randomized trial demonstrate that both DEM and DEX used as perineural adjuvants in ESPBs effectively prolong the time to first rescue opioid analgesia and reduce total opioid consumption in pediatric patients undergoing scoliosis surgery. These results highlight the potential benefits of incorporating these adjuvants into multimodal analgesia strategies for pediatric scoliosis surgical patients.

### Prolongation of Analgesia and Reduction in Opioid Consumption

One of the primary outcomes, the time to first rescue opioid analgesia, was significantly prolonged in both the DEX and DEM groups compared with the control group, indicating that both agents enhance the duration of analgesia provided by the ESPB. These findings are consistent with existing literature, where DEM^14^ and DEX^15^ have been shown to prolong the analgesic effect of regional anesthesia by different mechanisms—DEM through its α2-adrenergic agonist action,^16^ and DEX as it reduces ectopic neuronal discharge, inhibits potassium channel-mediated firing of nociceptive C fibers, and attenuates the release of inflammatory mediators.^15^

The significant reduction in total opioid consumption (in terms of intravenous morphine equivalents) in the DEX and DEM groups compared with the control group further supports the efficacy of these adjuvants in the pediatric population, especially considering the small number of studies on pediatric patients.^17–20^ Reduced opioid use is essential in the pediatric population, as it minimizes the risk of opioid-related adverse effects, including respiratory depression, nausea, and vomiting, and reduces the potential for opioid dependency.^21^ The comparable results between the DEX and DEM groups suggest that both agents are similarly effective in reducing opioid requirements, offering flexibility in clinical practice depending on patient needs or clinician preference.^14^

### Pain Scores and Clinical Implications

The pain scores (NRS) recorded postoperatively were significantly lower in the DEX and DEM groups at several time points, especially between 8 and 24 hours postsurgery. This sustained pain relief suggests that adding these adjuvants to the ESPB improves postoperative comfort during critical recovery. Interestingly, the NRS scores were comparable between the DEX and DEM groups, indicating that both agents provide similar levels of pain control, which is consistent with the literature.^14^ However, it is worth noting that the NRS scores at 4 hours postoperatively did not differ between the groups. This may indicate that the initial block effect of ropivacaine alone was sufficient to provide immediate postoperative analgesia in all groups. This highlights the importance of the adjuvants in prolonging analgesia rather than enhancing the initial block. However, there needs to be more evidence from the pediatric population, particularly in spinal surgeries, where comprehensive studies are scarce. This gap in research limits our understanding of the specific analgesic needs, safety profiles, and potential complications associated with different interventions in children undergoing these complex procedures. Further investigation is needed to establish clear guidelines and optimize pain management strategies for this vulnerable group.

### Hemodynamic Stability and Adverse Effects

DEM, widely recognized for its sedative and sympatholytic properties, demonstrated an incidence of bradycardia in 11 patients and hypotension in five patients within the DEM group during surgery, raising essential considerations regarding its intraoperative safety profile. In contrast, no hemodynamic disturbances were observed in the other groups, aligning with previous studies that consistently report DEM-induced hemodynamic alterations in children, specifically bradycardia and hypotension.^18,22^ These observations reaffirm the well-established cardiovascular effects of α2-adrenergic agonists, as bradycardia and hypotension are well-documented adverse effects of this drug class.^23^ Clinicians must remain vigilant in monitoring hemodynamics when administering DEM, particularly in pediatric populations, where the balance between therapeutic benefit and adverse effects is crucial.^18^ Although these events were effectively managed in our study, the findings underscore the necessity of careful hemodynamic surveillance during DEM use. Our results are consistent with the existing literature, further validating these hemodynamic concerns in pediatric and adult populations.^23–25^

The absence of nausea and vomiting in all three groups is a positive finding, particularly in the context of opioid-sparing analgesia. These results further reinforce the role of DEX and DEM in enhancing patient recovery by reducing common opioid-related side effects.

### Inflammatory and Metabolic Responses

In our study, no significant differences were observed between the groups regarding NLR, PLR, or blood glucose levels at any time. These findings suggest that DEM and DEX, used as perineural adjuvants in the ESPB, do not induce significant systemic inflammatory responses or metabolic disturbances during idiopathic scoliosis surgery. The stability of NLR and PLR further supports the notion that neither adjuvant significantly impacts the immune-inflammatory axis in this setting. Our findings are consistent with previous studies regarding the influence of peripheral nerve blocks^4,26,27^ and perineural adjuvants^15^ on systemic inflammatory response to surgery. In addition, the absence of significant variations in blood glucose levels between the groups indicates that DEX at the doses administered did not result in hyperglycemia, a common concern with corticosteroid use, consistent with the previous studies regarding children and adults.^15,28^

### Nerve Injury

Furthermore, no nerve damage was observed in any groups, suggesting that DEM and DEX are safe when administered perineurally in the context of ESPB for scoliosis surgery. This is particularly important in pediatric populations, where the risk of nerve injury must be minimized.^29^ These findings contribute to the growing body of evidence supporting the safety profile of both adjuvants in regional anesthesia and highlight their suitability for use in procedures requiring prolonged analgesia, such as spinal surgeries.^30^

### Neuromonitoring and MEPs

Effective MEP monitoring during surgery requires a comprehensive understanding of how anesthetic agents and physiological factors influence MEP signals. Optimizing neuromonitoring sensitivity depends on the appropriate selection and administration of anesthetic drugs.^10^ Notably, the DEX group exhibited the highest MEP amplitude at the T1 time point, suggesting that DEX exerts a lesser inhibitory effect on neuromonitoring parameters when used as an adjuvant than DEM.^31–33^ This finding is particularly relevant in procedures where intraoperative neuromonitoring is essential, such as spinal surgery, where MEP recordings serve as critical indicators of spinal cord integrity. In contrast, the relatively lower MEP amplitude observed in the DEM group may be attributed to DEM’s known sedative effects. However, the precise impact of these anesthetic adjuvants on neuromonitoring outcomes remains incompletely understood, highlighting the need for further research to elucidate their mechanisms and optimize intraoperative monitoring strategies.

### Limitations and Future Directions

Despite the significant findings, this study has several limitations. First, the sample size, though adequate for demonstrating noninferiority, may not be large enough to detect rare adverse events or subtle differences between DEM and DEX. In addition, the follow-up period was limited to 24 to 48 hours postoperatively, and longer-term outcomes, such as the development of chronic pain or prolonged recovery times, were not assessed. Future studies with larger sample sizes and extended follow-up are necessary to confirm these findings and further explore the long-term effects of using these adjuvants in pediatric surgery.

Another potential limitation is the exclusion of patients with more complex surgical needs or comorbidities, which may limit the generalizability of the results to all pediatric surgical patients. Future research should include a broader range of patients to evaluate the effects of these adjuvants in more heterogeneous populations.

## CONCLUSIONS

Both adjuvants, DEX and DEM, contribute to enhanced analgesia and reduced opioid requirements in pediatric scoliosis surgery. However, their distinct pharmacological effects on neuromonitoring and hemodynamic stability must be carefully considered when selecting an optimal adjuvant.

DEX provides superior neuromonitoring conditions, as evidenced by significantly higher MEP amplitudes and a reduced need for TES intensity.

In contrast, DEM, while effective in improving analgesia and minimizing opioid consumption, is associated with a pronounced suppression of MEP signals. This suppression can complicate intraoperative spinal cord monitoring and may require adjustments in neuromonitoring techniques to maintain surgical safety. Moreover, DEM’s sedative and sympatholytic effects contribute to greater hemodynamic instability, including bradycardia and hypotension, necessitating close cardiovascular monitoring and intervention when required.

These findings underscore the need for a balanced approach when integrating adjuvants into anesthetic protocols for pediatric scoliosis surgery. While both agents provide adequate analgesia, DEX is the preferred choice when neuromonitoring integrity is a primary concern. Future research should explore optimized dosing strategies and potential combination regimens to maximize the benefits of analgesia and neuromonitoring stability while minimizing hemodynamic side effects.

Key Points

- Impact on neuromonitoring:
  1. Dexamethasone enhances motor-evoked potential (MEP) amplitudes and reduces transcranial electrical stimulation (TES) intensity requirements, improving intraoperative neuromonitoring conditions.
  2. Dexmedetomidine suppresses MEP amplitudes and increases the risk of bradycardia, potentially compromising neuromonitoring reliability.
- Analgesic efficacy:
  1. Both dexamethasone and dexmedetomidine as ESPB adjuvants significantly prolong postoperative analgesia and reduce opioid consumption compared with ropivacaine alone.
  2. Pain scores at 8, 12, 16, and 24 hours were significantly lower in adjuvant groups.
- Hemodynamic effects:
  1. Dexmedetomidine was associated with bradycardia in 11 patients, whereas dexamethasone caused intraoperative hypotension in five patients, highlighting the need for careful hemodynamic monitoring.
- Clinical implications:
  1. Dexamethasone is preferable when optimizing both analgesia and neuromonitoring conditions in pediatric scoliosis surgery.
  2. Dexmedetomidine may be less suitable due to its suppressive effects on neuromonitoring and hemodynamic instability.
- Future considerations:
  1. Further studies should explore optimal dosing strategies and alternative adjuvants to maximize both analgesia and neuromonitoring integrity in scoliosis surgery.
